# Medical student research opportunities: a survey of osteopathic medical schools in the United States

**DOI:** 10.1515/jom-2021-0242

**Published:** 2022-03-02

**Authors:** Tyler Hamby, Don P. Wilson, Priya Bui, Jonathan Lowery, Riyaz Basha

**Affiliations:** Department of Research Operations, Cook Children’s Health Care System, Fort Worth, TX, USA; Texas College of Osteopathic Medicine at The University of North Texas Health Science Center, Fort Worth, TX, USA; Department of Pediatric Endocrinology and Diabetes, Cook Children’s Health Care System, Fort Worth, TX, USA; College of Osteopathic Medicine at Marian University, Indianapolis, IN, USA

**Keywords:** colleges of osteopathic medicine, medical education, medical student research, survey study

## Abstract

**Context:**

It is important for colleges of osteopathic medicine (COMs) to provide opportunities for osteopathic medical students (OMSs) to conduct research under the guidance of professional researchers. However, COMs historically lag behind allopathic medical schools in research offerings for medical students. The literature would benefit from a synopsis of research opportunities for OMSs at COMs.

**Objectives:**

This study aims to assess the availability of research opportunities currently offered to OMSs and to identify structured research programs (SRPs) to provide data that may help COMs expand such opportunities.

**Methods:**

Two online surveys were developed. The General Survey asked about general research opportunities, research requirements, and SRPs, which we define as optional, intramural, and mentored research programs. The follow-up SRP Survey sought to understand the history, funding, and organizational structure of SRPs. Between February and June 2021, the General and SRP Surveys were sent to all COMs in the United States. Response data were analyzed descriptively.

**Results:**

Responses were received from 32 (84.2%) of 38 COMs. Nearly all COMs offered research symposia, offered third- or fourth-year research elective rotations, and provided some form of funding for OMSs to participate in research. Fourteen (43.8%) COMs had mandatory research requirements. Twenty COMs (62.5%) offered 31 SRPs, and surveys were completed for 25 (80.6%) SRPs. SRPs were founded a median (range) of 7 (1–43) years prior and accommodated 20 (4–50) OMSs annually. Among the responding SRPs, 12.0% had external funding, 96.0% required applications, 50.0% interviewed applicants prior to acceptance into the program, 72.0% required OMSs to identify their own mentors, 68.0% offered stipends to OMSs, 28.0% offered course credits, 96.0% had clinical research opportunities, and 68.0% offered research-oriented didactics. In 84.0% of SRPs, OMSs worked predominantly in the summer after OMS-I; for these SRPs, students had 4–10 weeks of dedicated time for participation in research.

**Conclusions:**

Findings from our surveys provide a synopsis of the research opportunities currently provided by COMs in the United States. Our data demonstrated wide variability of research opportunities among COMs.

Several studies have indicated that conducting research during medical school improves students’ medical writing, ability to critically evaluate the literature, and knowledge of and skills for research processes [1]. The declining proportion of physician-scientists provides another reason to provide medical students with research opportunities [2], and a meta-analysis of three studies showed that research exposure in medical school increased medical students’ interest in conducting research in their future careers [3]. More specific to colleges of osteopathic medicine (COMs), the adoption of a single accreditation system for all medical students in the United States, which was implemented in 2020, places osteopathic medical students (OMSs) from COMs in direct competition with medical students from US allopathic medical schools (USMD) for residencies [4]. Because residency programs often consider publications and research experience to be important credentials when selecting residencies, it is more important than ever that COMs provide research opportunities to their OMSs [5]. Indeed, a study of 2016 and 2018 National Resident Matching Program data showed that medical students who matched in their preferred specialties had significantly more research accomplishments (abstracts, presentations, and publications) on average than did medical students who did not match in their preferred specialties. Unfortunately, the study also showed that COM graduates had significantly fewer research accomplishments on average than USMD graduates [6].

Opportunities for COMs to encourage student research include funding research expenses, organizing research symposia, adding mandatory research requirements to curricula, or offering third- or fourth-year research rotations. They may also offer dual-degree programs that require research, although these attract only a small proportion of the OMS population due to the heavy time and financial investments involved. Alternatively, COMs may offer structured research programs (SRPs), which we define as optional, extracurricular programs that allow OMSs to conduct research under mentors. Developing SRPs may be challenging for faculty and staff at COMs, owing to COMs’ relative lack of research infrastructure [4, 7]. Descriptive examples of SRPs may serve as models for individuals who wish to develop an SRP. However, only a few articles have been published that describe COM SRPs [8], [9], [10].

In the present study, we surveyed all US COMs about the research opportunities being provided to OMSs. The purpose of this study is to assess the present state of research opportunities being offered to OMSs.

## Methods

### Survey development

An online survey (Supplemental Material) was developed by a member of the study team (T.H.) in consultation with five faculty and staff members who served as a focus group. These faculty and staff included two PhDs and two MDs from a COM and a nonprofit, nonacademic, nonteaching children’s hospital. They included an oncologist and chair of a pediatrics department at the COM, an endocrinologist and director of medical education at the hospital, a biomedical sciences associate professor and vice chair for research at the COM, a research director at the hospital, and a research coordinator at the COM. The focus group discussed relevant survey topics and reviewed potential survey items iteratively, which enhanced content validity and face validity.

The study consisted of two separate surveys (Supplementary Material): the General Survey and the SRP Survey. Each survey began by defining SRPs as being optional intramural programs that allow OMSs to conduct research under mentors, and it was explained that elective research rotations and ad hoc research should not be included. The General Survey contained items concerning the presence of and contacts for an SRP, and the presence of other mandatory and optional research opportunities for OMSs. The SRP Survey asked respondents to confirm whether the program is an SRP, and it contains items about the SRP’s history, funding, and organization. Each COM completed the General Survey once at most, but the SRP Survey was conducted for each SRP listed in responses to the General Survey. The 9-item General Survey and 24-item SRP Survey each included checklist, categorical multiple-choice, and free-response item formats. Typically, the General Survey took less than 10 min to complete, and the SRP Survey took 10–15 min to complete.

### Participants

All 43 COMs listed in the 2020–2021 American Association of Colleges of Osteopathic Medicine (AACOM) Student Guide to Osteopathic Medical Colleges were surveyed [11]. For the General Survey, contact information was garnered from the websites for COMs; potential respondents were generally contacted in the following sequence: (1) research directors; (2) deans of research; and (3) COM deans. For the SRP Survey, potential respondents included those provided by respondents to the General Survey, but other potential respondents associated with the SRP were identified on websites for COMs as needed. Survey completion was voluntary, and no financial compensation or other incentives were provided for respondents.

### Data collection

The online survey was developed in and administered via REDCap, which housed the responses in a secure database. Links to the REDCap surveys were provided in emails to potential respondents [12]. A single potential respondent was emailed up to 3 times over 3 weeks for each survey. If no response was received after 4 weeks, this process was repeated with another potential respondent twice more for each General Survey and once more for each SRP Survey. General Surveys were emailed between February and May 2021, and SRP Surveys were emailed between April and June 2021. After the General Survey was completed, the COM’s SRP Survey(s) was emailed simultaneously with the next set of initial emails to potential respondents beginning in April. Each survey could be taken only once and was closed after completion. Data collection was complete in June 2021. Attempts were made to clarify missing or ambiguous responses via email.

### Data analysis

For COMs with multiple affiliated campuses (e.g., A.T. Still University COM, Edward Via COM), each campus was sent the General Survey and then the responses were compared. If the responses were similar across campuses, they were combined into one COM; otherwise, each campus was reported separately. When estimates were given for the year that the SRP was developed (e.g., “before 2003,” “prior to 2015”), the most recent year was utilized (e.g., 2003, 2015). When ranges were provided for the average number of students in the SRP per year (e.g., “30–40 students”), the average value was utilized (e.g., 35). Responses to the quantitative items were described with frequencies and percentages for categorical variables; medians and ranges were utilized for numerical variables because they were skewed and non-normally distributed. Relationships between numerical variables were examined with Pearson’s correlation. Data were analyzed in SAS Enterprise (version 6.1; SAS Institute Inc, Cary, NC). Graphs were developed utilizing RStudio (RStudio, Inc, Boston, MA). This study was approved by the Cook Children’s Health Care System Institutional Review Board (IRB) as exempt and completing the survey’s implied consent.

## Results

### General Survey

There was a total of 43 COMs surveyed: 27 single-campus COMs; three COMs with two campuses; two COMs with three campuses; and one COM with four campuses. Two multi-campus COMs were identified as providing similar research opportunities across campuses, and responses were combined for both COMs. Of the remaining 38 COMs, 32 (84.2%) responses were received. Table 1 summarizes the results of the General Survey.

**Table 1:** Results of general survey (n=32).

Item	n (%)
Research day or symposium	32 (100.0%)
Funding: travel for presentations	29 (90.6%)
Funding: printing posters	29 (90.6%)
Funding: research projects	24 (75.0%)
Funding: publication costs	22 (68.8%)
Elective research rotation	31 (96.9%)
Mandatory research requirements	14 (43.8%)
Structured research program(s)	20 (62.5%)

All COMs reported having a designated research day or symposium in which OMSs may present research. Most COMs provided OMSs with funding for travel for presentations (29 [90.6%]), costs for printing posters (29 [90.6%]), research projects (24 [75.0%]), and publication costs (22 [68.8%]). Almost all (31 [96.9%]) COMs, provided funding for one or more of the above.

Thirty-one (96.9%) COMs offered elective research rotations in OMS-III and/or OMS-IV. Only 14 (43.8%) COMS had mandatory research requirements for OMSs. Twenty-three (71.9%) COMs reported having a total of 41 SRPs. However, 10 (24.4%) SRPs were excluded from further analysis: one was a dual-degree program; one was an elective research rotation; and eight were later verified not to be SRPs by respondents. After these exclusions, 20 (62.5%) COMs reported having 31 SRPs: 13 (65.0%) COMs had one SRP; 4 (20.0%) COMS had two SRPs; 2 (10.0%) COMs had three SRPs; and 1 (5.0%) COM had four SRPs.

### SRP survey

**Figure 1:**
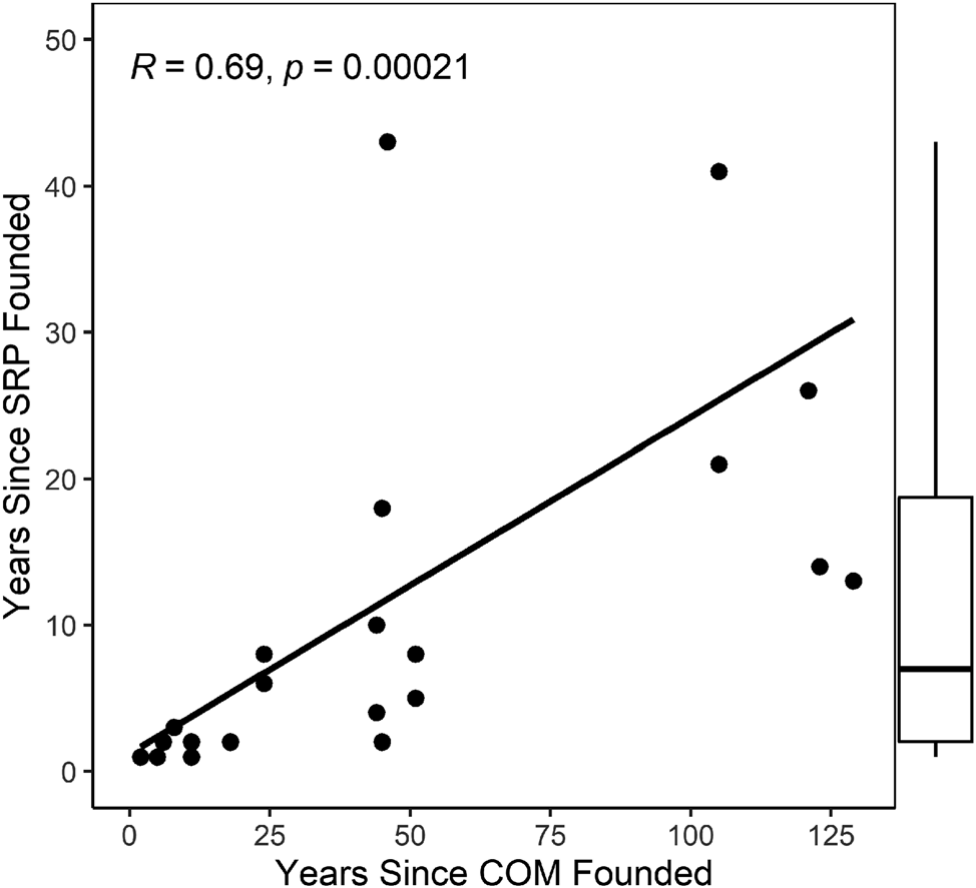
Scatterplot of association between years since the colleges of osteopathic medicine and structured research programs were founded. COM, college of osteopathic medicine; SRP, structured research program; *R*, Pearson’s correlation; *p*, *p*-value.

Responses were received for 25 (80.6%) of the 31 SRPs reported. Table 2 summarizes the results of the SRP Survey. These programs were founded a median (range) of 7 (1–43) years prior and all were ongoing. These SRPs have existed for a median (range) of 20.7% (4.4–93.5%) of their respective COMs’ existence [11]. There was a strong, significant correlation between the years since the COMs and the SRPs were founded (Figure 1). Only 3 (12.0%) SRPs from two COMs were supported by external funding. For OMSs to participate, they were required to submit applications for 24 (96.0%) SRPs and be interviewed for 12 (50.0%) SRPs. Students selected their own mentors for 18 (72.0%) SRPs and were assigned their mentors in 7 (28.0%) SRPs.

**Table 2:** Results of structured research program survey (n=25).

Item	n (%)
External funding	3 (12.0%)
Require student applications	24 (96.0%)
Require student interviews	12 (50.0%)
Students select mentors	18 (72.0%)
Course credit	7 (28.0%)
Stipends or financial support for some or all students	17 (68.0%)
Research option: clinical research	24 (96.0%)
Research option: public health, health services, and/or epidemiology	22 (88.0%)
Research option: basic or translational science	21 (84.0%)
Research option: ethics, humanities, and/or social science	15 (60.0%)
Didactic lectures on research	17 (68.0%)
Students work predominantly in summer after OMS I	21 (84.0%)

**Figure 2:**
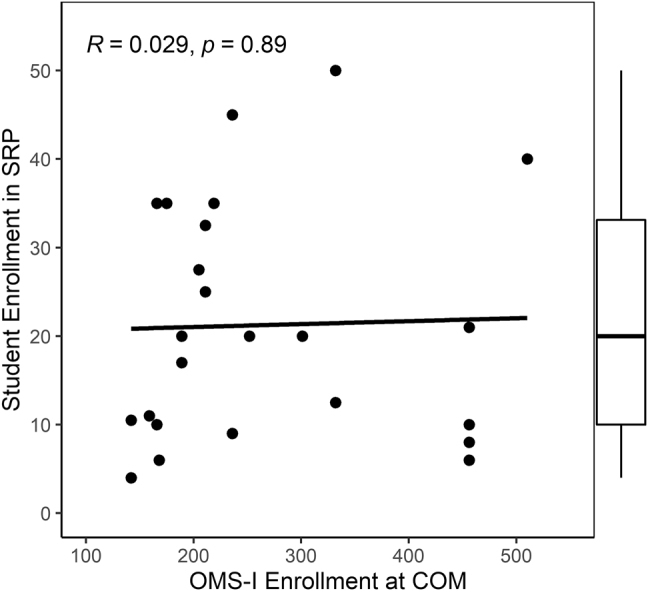
Scatterplot of association between enrollments at colleges of osteopathic medicine and in structured research programs each year. COM, college of osteopathic medicine; SRP, structured research program; *R*, Pearson’s correlation; *p*, *p*-value.

A median (range) of 20 (4–50) OMSs participated in the SRPs each year. Taken as proportions of 2019 OMS-I enrollment [13], these programs accommodated a median (range) of 7.6% (1.3–21.1%) of students enrolled per class. There was a negligible correlation between the numbers of participants in SRPs and enrollment in the corresponding COMs (Figure 2). Course credit was provided for only 7 (28.0%) SRPs. Eight (32.0%) SRPs did not provide stipends or financial support to students; the others provided funding for some (4 [16.0%]) or all (13 [52.0%]) students. The research opportunities offered included clinical research (24 [96.0%]); public health, health services, and/or epidemiology (22 [88.0%]); basic or translational science (21 [84.0%]); and ethics, humanities, and/or social sciences (15 [60.0%]). Seventeen (68.0%) programs provided didactic lectures on research.

For 21 (84.0%) SRPs, OMSs did most of their work in the summer after OMS-I, although these programs typically had requirements beyond the summer. Seventeen (68.0%) SRPs required that OMSs dedicate a median (range) of 7 (4–10) weeks to research in the summer: 4 weeks (1 [4.0%]), 6 weeks (7 [28.0%]), 7 weeks (1 [4.0%]), 8 weeks (6 [24.0%]), and 10 weeks (2 [8.0%]). One (4.0%) SRP permitted up to 240 h of work to be completed by the end of OMS-II, and 2 (8.0%) SRPs required that OMSs dedicate a specified number of hours (80 and 200) to research between the summer and a specified date in the following winter. Two (8.0%) SRPs had requirements for each year of medical school but specified neither the numbers of weeks dedicated in summer nor hours required.

For 4 (16.0%) SRPs, OMSs predominantly worked outside of the summer between OMS-I and OMS-II. One (4.0%) SRP required that OMSs dedicate 3 months to research any time from the summer after OMS-I to graduation, 1 (4.0%) SRP required that OMSs dedicate 3–5 hours per week to research throughout OMS-II, and 2 (8.0%) SRPs required that OMSs take a fifth year of training for research.

## Discussion

The present study provides, to our knowledge, the first overview of research opportunities being offered to OMSs at COMs across the United States; and with response rates >80% for both the General and SRP Surveys, the results are likely representative. The data on structural characteristics of the SRPs may prove to be useful to faculty and staff at COMs who seek to develop an SRP. Administrators may select the SRP characteristics that fit best with their COMs’ administrative capacity, faculty, and student curricula.

In general, the results show that US COMs provided a variety of research opportunities for OMSs. All COMs sponsored annual research symposia to allow students to present their research findings, and nearly all COMs offered elective research rotations during OMSs’ clerkship years. Although the numbers differed by category, nearly all COMs funded OMSs for at least one research activity.

The proportion of COMs with mandatory research requirements was comparable to that reported in 2017 for USMDs (43.8 vs. 44.2%) [14]. However, survey items were not included to determine what COMs required students to do, and the free-text descriptions of these requirements showed great variability between COMs. There are benefits of requiring all OMSs to conduct research. For example, some OMSs, who may not have opted for voluntary research experiences, may discover that they enjoy research. Additionally, all OMSs can benefit from learning those research skills (e.g., literature review, hypothesis generation) that have applications to clinical practice, and research experiences improve OMSs’ resumes for residencies [15]. However, a survey study of OMS-I students at four COMs suggests that not all OMSs (177/328 [54.0%]) are interested in research [16], and medical students have been shown to report greater satisfaction with voluntary, compared to mandatory, research programs [17]. Further, requiring disinterested OMSs to conduct research under faculty mentorship is a drain on financial resources and faculty members’ time [15].

Among the numerous options for voluntary research programs, dual-degree PhD programs provide the most rigorous training for prospective clinician-scientists. Recent data show that only 18.6% of COMs [18], compared with 71.0% of USMDs [19], offer dual-degree PhD programs. In light of the obstacles that COMs face in providing research opportunities to OMSs [4, 7], voluntary SRPs may be more appropriate than either curricular research requirements or PhD programs.

Encouragingly, most COMs offered at least one SRP. The finding that 33.3% of SRPs were developed in the prior 2 years suggests a recent push by COMs to provide OMSs with research opportunities. Still, most SRPs had been active for ≥5 years, which speaks to these programs’ sustainability. Critically, 90% of COMs with SRPs supported these programs without extramural funding.

Most SRPs provided some or all OMSs with stipends, and some programs offered course credit. Although a survey of OMS-I students at four COMs showed both monetary compensation (213/328 [64.9%]) and extra credit in courses (195/328 [59.5%]) to be strong incentives for OMSs to participate in research, the same study also showed that a slim majority of respondents were either currently doing research or planning to do research while in medical school [16]. In the present study, seven SRPs provided neither incentive, including the three SRPs with the highest proportions of participating students relative to total enrollment. Still, in unreported analyses, neither the number of SRP participants nor the SRP student participation as a proportion of annual enrollment statistically significantly differed based on whether either incentive was offered. The effects of financial and course credit incentives on participation in SRPs should be empirically examined. If students are willing to participate in SRPs without incentives, those resources may be more efficiently utilized to provide administrative and research support for SRPs.

Nearly all SRPs required students to apply to participate, but only half interviewed applicants. Most SRPs had students select mentors. Interestingly, of the seven SRPs that assigned mentors to students, only one SRP—the Pediatric Research Program, which some of the authors oversee (T.H., D.P.W., P.B., and R.B.)—interviewed applicants. In our experience, interviews provide an opportunity to probe further into students’ interests and to determine compatibility with potential mentors and projects. Nearly all SRPs offered clinical research options, which 3 prior survey studies have shown to be the most popular type of research for OMSs (65.5–82.0%) [16, 20, 21]. A majority of SRPs provided research lectures or didactics. Although most SRPs were longitudinal to some extent, the typical SRP had students work primarily in a 4- to 10-week period in the summer after OMS-I. However, some programs lasted 1 year or all 4 years.

Although numerous descriptions of SRPs at USMDs and some at COMs have been published, no study has systematically surveyed them for comparison. However, in one study, investigators surveyed all summer SRPs in Canadian medical schools and affiliated institutions; although the response rate was 50.5% and SRPs with only undergraduate students were included, the results may be compared [22]. Canadian summer SRPs had a much larger average number of students per year than COM SRPs (40.04 vs. 20.76). Compared to COMs’ SRPs, Canadian summer SRPs were more likely to have been active for ≥5 years (78.3 vs. 58.3%), were less likely to have clinical opportunities (87.0 vs. 96.0%), and were similar in the likelihood of assigning students to mentors (81.8 vs. 72.0%) and providing didactic lessons (61.4 vs. 68.0%). Probably owing to the inclusion of undergraduate students with longer summer breaks, Canadian summer SRPs tended to have a greater duration in weeks than summer COM SRPs. Canadian summer SRPs were far more likely to have external funding—from private investigator grants (45.7%), private donations (43.5%), or government support (13.0%)—than were COM SRPs (12.0%).

### Limitations and directions for further research

Although the present results provide valuable information about research opportunities for OMSs at US COMs, limitations must be acknowledged. The primary limitation of the present study was that respondents may not have had all information required to complete the survey. A lack of readily available information may have dissuaded some potential respondents from participating. Lack of information and the social desirability bias could potentially have produced inaccurate responses. To address these concerns, survey responses were compared with information from respective COMs’ websites when available, although survey responses were unaltered when inconsistencies were noted. Overall, inconsistencies between responses and COMs’ websites were rare. Importantly, respondents were asked not to submit the survey until all information was verified and to forward the survey to more appropriate respondents if applicable; therefore, we believe inaccurate responses to be minimal.

The present study offers a broad overview of research programs for OMS at COMs, and the surveys were purposefully brief to achieve a high response rate. Although a high response rate was achieved, the resulting data are limited in depth. Perhaps a lengthier survey conducted under the auspices of the AACOM would produce more detailed data while still retaining a high response rate. The General Survey provided no quantitative data about research symposia, research funding, elective research rotations, and mandatory research requirements beyond their presence or absence. A direction for further research would be to conduct a more detailed survey study focusing on these questions. The SRP Survey provided more detailed quantitative data on SRPs, but it lacked the qualitative data necessary to understand the practical management of SRPs. It is important that more researchers publish their COMs’ experiences with SRPs. Additionally, it would be useful to conduct a multicenter study that contrasts several SRPs both quantitatively and qualitatively. Such studies provide usable templates for faculty and staff at COMs to develop research opportunities for OMSs. Lastly, it would be interesting to conduct a survey of research opportunities for medical students at all USMDs and COMs to allow for comparisons.

## Conclusions

The present survey study provides a generalizable overview of the research opportunities that US COMs currently offer OMSs. Results demonstrated the variability of research opportunities among COMs. All COMs attempted to provide their OMSs with research opportunities. Most COMs offered SRPs, and there is evidence of a recent push to expand these opportunities. The majority of SRPs took place in the summer after OMS-I.

## Supplementary Material

Supplementary Material
