# Supplementary material for: Medical student research opportunities: a survey of osteopathic medical schools in the United States
Source: J Osteopath Med. 2022 Mar 2;122(6):289–95. doi: 10.1515/jom-2021-0242 (PMC9131179; doi:10.1515/jom-2021-0242)
Supplement: Supplementary file 1 — Supplementary Material [file j_jom-2021-0242_suppl.docx]

**Appendix.**

**General Research Survey**

Name of college of osteopathic medicine

________________________________________________________________

Name and email address of person completing this form

________________________________________________________________

For the following questions, we will refer to "structured research programs" as programs that:

(1) allow medical students to conduct research under mentors

(2) are optional

(3) are intramural

Typically, structured research programs will require students to apply and will have start and end dates.

For structured research programs, please do NOT include 3rd- or 4th-year elective research rotations. Additionally, many schools permit students to conduct ad hoc research with faculty outside the context of a program; we do NOT consider these opportunities to be structured research programs.

Does your school have any current or former structured research programs?

- Yes
- No

For each structured research program, please provide the name of the program and contact information

________________________________________________________________

Does your school organize a research day or research symposium for medical students to present their research?

- Yes
- No

Does your school provide medical students with funding for the following? (check all that apply)

- Research Projects
- Travel Expenses for Presentations
- Costs for Printing Posters
- Publication Costs

Does your school allow 3rd- and/or 4th-year medical students to take elective research rotations for course credit?

- Yes
- No

Does your school have any mandatory research requirements for medical students?

- Yes
- No

Please describe any mandatory research requirements for medical students.

________________________________________________________________

**Structured Research Program Survey**

Name and email address of person completing this form

________________________________________________________________

Name of college of osteopathic medicine

________________________________________________________________

For the following questions, we will refer to "structured research programs" as programs that:

(1) allow medical students to conduct research under mentors

(2) are optional

(3) are intramural

Typically, structured research programs will require students to apply and will have start and end dates.

For structured research programs, please do NOT include 3rd- or 4th-year elective research rotations. Additionally, many schools permit students to conduct ad hoc research with faculty outside the context of a program; we do NOT consider these opportunities to be structured research programs.

Program name:

________________________________________________________________

Is or (if the program was discontinued) was the above program a structured research program?

- Yes
- No

What year did this program begin?

________________________________________________________________

Is this program ongoing or has it been discontinued?

- Ongoing
- Discontinued

When was the program discontinued?

________________________________________________________________

Why was the program discontinued?

________________________________________________________________

Is (was) the program supported by intramural and/or extramural funding?

- Intramural funding
- Extramural funding

What is (was) the funding agency?

________________________________________________________________

Do (did) medical students apply to be in the program?

- Yes
- No

Are (were) medical students interviewed before being selected?

- Yes
- No

Typically, do (did) medical students find and select their mentor or are (were) students assigned mentors?

- Students select(ed) mentors
- Students are (were) assigned mentors

Please indicate which types of research are (were) permitted:

- Basic or translational science
- Clinical research
- Public health/health services/epidemiology
- Ethics/humanities/social sciences

Do (did) students do most of their work for this program in the summer between their 1st and 2nd years of medical school?

- Yes
- No

Do (did) medical students take an extra year to dedicate to this program?

- Yes
- No

When do (did) students typically conduct most of their research for this program? (check all that apply)

- OMS-I
- OMS-II
- OMS-III
- OMS-IV

At minimum, how long are (were) medical students expected to work on research for this program?

________________________________________________________________

Does (did) this program provide didactics or lectures on research?

- Yes
- No

Approximately how many medical students participate(d) in this program each year?

________________________________________________________________

Do (did) medical students receive course credit for this program?

- Yes
- No

Are (were) all, some, or none of the medical students provided a stipend or financial support?

- All
- Some
- None

What are (were) the goals for this program?

________________________________________________________________

What are (were) the expectations for this program?

________________________________________________________________
